# Editorial: Reproductive health and well-being from a life span perspective

**DOI:** 10.3389/fpsyg.2023.1289603

**Published:** 2023-10-26

**Authors:** Karolina Lutkiewicz, Łucja Bieleninik, Dian Veronika Sakti Kaloeti, Mariola Bidzan

**Affiliations:** ^1^Department of Clinical Psychology and Health, Faculty of Social Sciences, Institute of Psychology, University of Gdańsk, Gdańsk, Poland; ^2^GAMUT-The Grieg Academy Music Therapy Research Centre, NORCE Norwegian Research Centre, Bergen, Norway; ^3^Faculty of Psychology, Universitas Diponegoro, Semarang, Indonesia

**Keywords:** reproductive health, wellbeing, life span perspective, infertility, pregnancy

According to the World Health Organization reproductive health contributes to mental, social and physical wellbeing. This Research Topic focuses on wellbeing and mental health using the life span approach to address the psychological difficulties of individuals, couples, and families that can be experienced in reproductive health. Taking a life span perspective in researching reproductive health includes investigating various factors that may influence reproductive processes, functions, and systems across different stages of life (Mishra et al., 2010). Moreover, the life span approach in studying reproductive health is gaining much more attention. It is being used to study biological, physical, behavioral, social, and cultural factors during gestation, childhood, adolescence, and adulthood that affect reproductive health. It also provides a more comprehensive vision of reproductive health and its risk factors across previous generations related to the menstrual issues, fertility, infertility, pregnancy, gynecological problems, abortion, miscarriages, menopause, and risk of chronic diseases (Kuh et al., 2003; Mishra et al., 2010). The life span perspective offers a framework that investigates opportunities to improve health in later life and highlights the importance of services that focus on the needs of individuals/groups in critical periods of life. It is worth emphasizing that the life-span approach was related to the revision of the concept of development, reformulating the subject of research and creating a function for a pluralistic approach in the research of human development (Trempała, 2001). Psychologists (including Paul Baltes) became interested in human development throughout life, developmental transformations of the adult psyche, along with old age. To distinguish from the traditional approach, developmental psychology practiced according to a new formula and was called “human development psychology”, “developmental psychology in the sequence of life” or “the psychology of human development throughout life” (Baltes, 1987, 1997; Stuart-Hamilton, 2006; Finogenow, 2013). According to the principles of life-span psychology, development is a lifetime process that lasts from birth to death. It is a multi-dimensional, multidirectional and contextual process, which encompasses both progressive and regressive changes (Baltes, 1987; Finogenow, 2013). Using the integrated life span perspective to research reproductive health emphasizes the continuity and multidimensionality of health. It focuses on the interrelationships of different elements related to reproductive health rather than examining risk factors separately (Mishra et al., 2010).

The following Research Topic gathers knowledge about reproductive health. It is focused on adults and their experienced pregnancy, birth trauma and infertility.

Pregnancy and the first year of postpartum is a vulnerable time for parents and the whole family system. A study conducted by Qin et al. “*Characteristics and related factors in Chinese females and partners during early pregnancy”* aimed to explore the characteristics of family functioning while promoting a basic understanding of family functioning among pregnant women and their partners in early pregnancy. Various research has previously focused on the impact of impaired family functioning in general, but only a limited number of studies have explored family functioning in the vulnerable period such as early pregnancy. The perinatal period is crucial for mitigating stress, enhancing coping abilities, averting depression, and adapting to the new maternal role during pregnancy (McLeish and Redshaw, 2017; Tang et al., 2019). Therefore, the study conducted by Qin et al. showed that early pregnancy is a vulnerable period for family functioning, where early-pregnancy females and their partners with poor family functioning of behavior control showed more severe depressive and anxious symptoms. These findings pay attention to the need for psycho-educational and support programs for families in early pregnancy to raise clinical awareness of the importance of family health during this sensitive period.

Childbirth can be considered as one of the most important events in a woman's life (Okay and Yavuz Güler, 2021; Shorey and Wong, 2022). However, negative birth experiences can also cause psychological trauma to women (Fenech and Thomson, 2015; Shorey and Wong, 2022; Chrzan-Dętkoś and Murawska, 2023; Sun et al.). Studies indicate that the incidence of traumatic birth ranges from 20 to as high as 68.6 percent in different countries (Bay and Sayiner, 2021; Sun et al.). The research results show that trauma during childbirth can translate into the poor mental health of the women themselves. It also can cause a deterioration in parent-infant relationships, breast feeding behavior, marital relationships, and future reproductive decisions (Bielawska-Batorowicz, 2006; Bieleninik et al., 2009; Taghizadeh et al., 2013; Holopainen et al., 2020; Sun et al.). Factors that increase the risk of post-partum trauma in women encompass experiencing complications with one's birth plan, giving birth to a baby with health issues or abnormalities, and being subjected to verbal abuse (Liu et al., 2021; Martinez-Vázquez et al., 2021). A review conducted by Sun et al. *Psychological birth trauma: A concept analysis* attempted to deepen the understanding of the concept of psychological birth trauma by using comprehensive concept analysis. The authors defined psychological birth trauma as the “woman's subjective feeling caused by events directly or indirectly related to childbirth, which is manifested as intertwined painful emotional experiences that originate in the birth process and last until postpartum” (Sun et al.). This literature review highlights the importance of prevention, identification, and intervention to prevent the negative effects of psychological birth trauma, which is still limited in research studies. Overall, the emotion-focused approach to prevent psychological birth trauma has received more attention as it has shown more effectiveness among primiparas women. However, the integration of this approach in health programs is challenging due to content, duration, frequency and timing of these interventions. This review provides a crucial starting point in psychological birth trauma both for theory and research as well as the clinical implications for the development of screening tools and designing appropriate interventions for those who experience psychological birth trauma (Sun et al.).

Many researchers have examined parental bonding over the last few decades since the concept was initially described in the 1970s (Klaus et al., 1972). The process of forming a healthy emotional tie between parents and their newborn baby in the early post-partum period is crucial due to its long-lasting impact on the future parent–infant attachment (Mihelic et al., 2017; Nelson et al., 2019), the child's survival and consequently the child's development (Leckman et al., 2004; Chrzan-Dętkoś et al., 2014; Nakano et al., 2019; Lutkiewicz et al., 2020; Bieleninik et al., 2021). In the study conducted by Bohne et al.: *Do parental cognitions during pregnancy predict bonding after birth in a low-risk sample?* the authors assessed the influence of cognitive factors on bonding with a child after delivery. Results indicated that the mothers' relationship with the infant is essential for how they experience their baby, while fathers reported their own wellbeing as being the most important factor that impacts their bonding with a child (Bohne et al.). Health care programs should be implemented to screen not only parental mental health, but also vulnerable parents who may have trouble to grow an emotional tie with their baby starting in pregnancy to help them facilitate optimal bonding after birth. The authors suggested that identifying repetitive negative thoughts during pregnancy and helping reduce these thoughts might facilitate stronger bonding with the infant and therefore be a better start for the family (Bohne et al.).

Having a child is accepted as a psychological, biological, social, and cultural need for almost every community. Due to cultural expectations, couples think about how many children they want and when they want them instead of considering whether they can have a child (Bidzan, 2010; Siyez et al., 2018). Pregnancy is viewed as a component of a women's wellbeing and a way to manifest their maternal instincts (Van den Heuvel, 2022). Furthermore, within Eastern cultures, infertility is regarded as shameful and frequently linked to societal myths, some of which involve unsettling supernatural forces (Ali et al., 2011). This situation can affect the partner's mental wellbeing, highlighting the necessity for support from diverse sources, with a particular emphasis on family (Lam et al., 2021). Nevertheless, not all couples have children. One factor contributing to this issue is the choice to abstain from having children, often called being child-free (Nakkerud, 2021). This phenomenon is on the rise and is closely tied to people's attitudes toward reproduction (Nakkerud, 2021). Childfree couples hold the perspective that assuming the role of a parent is a conscious decision, encompassing not only fulfillment but also the challenges associated with parenthood (Bhambhani and Inbanathan, 2020). Particularly in developed nations, the increasing number of women engaged in careers and pursuing personal interests plays a significant role in choosing to lead a “child-free” life (Verniers, 2020). Women who opt not to have children perceive their decision as freedom in various aspects of life, although they may still experience natural maternal instincts, such as showing affection when interacting with young children (Parlak and Tekin, 2020). Despite research indicating no disparities in work attitudes and skill development between childfree couples and those with children, childfree couples often find themselves perceived as deviating from societal expectations (Ingalls, 2016). It's worth noting that further research is needed to comprehensively understand the impact of childfree couples' reproductive attitudes on their mental wellbeing.

One of the most researched reasons for not having children is infertility. Infertility is not a recent problem, as it has affected people since time immemorial (Bidzan, 2006, 2010; Bielawska-Batorowicz, 2006; Podolska, 2007; Podolska and Bidzan, 2011). The World Health Organization (WHO) estimates that about 10–25% of couples have fertility problems (Podolska and Bidzan, 2011; Vander Borght and Wyns, 2018). The intensity of procreation problems is manifesting an upward tendency and is becoming an important issue in the modern world. It is noteworthy that the incidence of infertility in women and men is similar (McDonald Evens, 2004; Podolska, 2007; Bidzan, 2010). In most cases, the relationship between partners becomes disturbed, manifested by conflicts, which lowers the self-esteem of women and increases stress. Sexual life becomes a monotonous routine, resulting in the incidence of secondary sexual intercourse disorders (Kentenich, 2002; Bidzan, 2010; Podolska and Bidzan, 2011). Moreover, infertility can also negatively impact a women's body image (Younesi and Salajegheh, 2001). In the context of infertility, the body appears to be simultaneously both what prevents women from conceiving and the target of medically assisted reproductive techniques (Cousineau and Domar, 2007; Cipolletta and Faccio, 2013). This includes procedures that can be physically and psychologically highly intrusive (Cousineau and Domar, 2007) and can have implications in terms of grief experiences (McBain and Reeves, 2019), such as the loss of bodily integrity and a loss of control over the body, as well as identity related issues (Bell, 2019). As a result, the authors became interested in protective factors of body image in people with infertility, such as attachment style.

Calvo et al. raised the issue regarding *Romantic attachment, infertility-related stress, and positive body image of women dealing with infertility*. Their findings suggest that romantic attachment insecurities and infertility-related stress are significantly associated with a worsened body image in infertile women. Some people treated for infertility also undergo assisted reproduction procedures, e.g., *in vitro*, which is a source of stress and elevated anxiety or even depression (Olivius et al., 2004). It is an experience, which requires additional coping efforts from the individual experiencing the treatment (Kroemeke and Kubicka, 2017).

Significant findings were also brought by the research article by Wu et al. *Psychological distress among women undergoing in vitro fertilization-embryo transfer IVF-ET: A cross-sectional and longitudinal network analysis*. This longitudinal study provides novel insights into the symptoms of depression and anxiety and its network structure among women undergoing IVF-ET treatment. The authors also investigated whether the network changed over the course of treatment by looking at the network structure patterns at each of the four cross-sectional time points separately, and by exploring the connections between symptoms in a longitudinal network considering changes in individual components over time (Wu et al.). Obtained results in this study suggest that the treatment of mental health problems experienced by women receiving IVF-ET may be directed to the symptoms highlighted by network analysis in this study. For example, interventions related to the mind and body, cognitive behavioral therapy, and brief mindfulness programs, targeting the inability to concentrate, inability to relax, and guilt are likely to have the potential to relieve patients from anxiety and depression symptoms (Bai et al., 2019). This is the first network analysis to focus on psychological distress during IVF-ET treatment in women, which offers an important contribution to the understanding of interactions and changes in depression and anxiety symptoms and meeting the mental health needs of women undergoing IVF-ET treatment.

Infertility literature suggests the widespread recourse to long-term medical treatments despite the evidence of high stress, costs, and the adverse effects of repeated treatment failures. However, there is a lack of research comparing predictors of stress and psychological health outcomes between members of infertile couples who—after repeated failures—persist in pursuing medical treatments (PT) with those who opted for quitting treatments and adopting (QTA). Based on a transactional and multi-dimensional approach to infertility-related stress and health, Zurlo et al. in the study *Paths toward parenthood after repeated treatment failures: a comparative study on predictors of psychological health outcomes in infertile couples persisting in treatments or opting for adoption* assessed individual (socio-demographic; coping strategies) and situational (infertility-related parameters; infertility-related stressors, couple's dyadic adjustment dimensions) predictors of state-anxiety and depression in infertile couples pursuing medical treatments (PT) and quitting treatments and adopting (QTA) infertile couples. The results indicated that members of infertile couples quitting treatments and adopting (QTA) reported significantly lower levels of state anxiety, depression and stress related to the need for parenthood and a rejection of the childfree lifestyle. This included lower pressure related to social and the couple's relationship concerns than those who persist in pursuing medical treatments (PT). They also showed that members of infertile couples quitting treatments and adopting (QTA) recurred to a greater extent to active coping strategies (problem-solving/social support) and to a lower capacity to passive coping strategies (avoiding/turning to religion), and they reported higher levels of dyadic adjustment. Specificities in the main and moderating factors related to state anxiety and depression by study groups and across genders have also been found (Zurlo et al.). This is another study that highlights the importance of tailored counseling including men and women struggling with the long and prolonged infertility treatments.

To conclude, problems related to reproductive health are complex, including both men and women. The presented articles included in this Research Topic indicates a need for further research on reproductive psychology, which relates to wellbeing and mental health throughout all stages of the reproductive process from the life span perspective. Future research is needed to focus on the screening and assessment of mental health, not only of those who are expecting a child but also those who struggle with infertility. Different strategies for detection, prevention and treatment are needed. Undertaken research has resulted in practical applications that can be used by specialists, primarily psychologists and psychotherapists, not only in psychoeducation but also in the offered psychological and psychotherapeutic help. Research indicates the need for screening tools and intervention programs, which are matched to individual needs, as essential for the help and treatment among various vulnerable groups of people. It is also important to acknowledge that studies on more diverse samples is needed. For example, inducing those with lower socioeconomic backgrounds, lower education, single parents and those who identify as lesbian, gay, bisexual, transgender and queer LGBTQ communities. All people need sexual and reproductive health care, thus promoting support interventions while coping with reproductive problems should be inclusive, open to different lifestyles and personal experiences. Across countries, there are still inequalities in the management of reproductive health, thus universal and evidence-based practice guidelines related to reproductive health are needed.

## Author contributions

KL: Writing—review & editing. ŁB: Writing—review & editing. DK: Writing—original draft. MB: Writing—original draft.
